# ESR paper on structured reporting in radiology—update 2023

**DOI:** 10.1186/s13244-023-01560-0

**Published:** 2023-11-23

**Authors:** European Society of Radiology (ESR), Daniel Pinto dos Santos, Elmar Kotter, Peter Mildenberger, Luis Martí-Bonmatí

**Affiliations:** European Society of Radiology (ESR), Am Gestade 1, Vienna, 1010 Austria

**Keywords:** Policy, Communication, Radiology information systems

## Abstract

Structured reporting in radiology continues to hold substantial potential to improve the quality of service provided to patients and referring physicians. Despite many physicians’ preference for structured reports and various efforts by radiological societies and some vendors, structured reporting has still not been widely adopted in clinical routine.

While in many countries national radiological societies have launched initiatives to further promote structured reporting, cross-institutional applications of report templates and incentives for usage of structured reporting are lacking. Various legislative measures have been taken in the USA and the European Union to promote interoperable data formats such as Fast Healthcare Interoperability Resources (FHIR) in the context of the EU Health Data Space (EHDS) which will certainly be relevant for the future of structured reporting. Lastly, recent advances in artificial intelligence and large language models may provide innovative and efficient approaches to integrate structured reporting more seamlessly into the radiologists’ workflow.

The ESR will remain committed to advancing structured reporting as a key component towards more value-based radiology. Practical solutions for structured reporting need to be provided by vendors. Policy makers should incentivize the usage of structured radiological reporting, especially in cross-institutional setting.

**Critical relevance statement** Over the past years, the benefits of structured reporting in radiology have been widely discussed and agreed upon; however, implementation in clinical routine is lacking due—policy makers should incentivize the usage of structured radiological reporting, especially in cross-institutional setting.

**Key points**

1. Various national societies have established initiatives for structured reporting in radiology.

2. Almost no monetary or structural incentives exist that favor structured reporting.

3. A consensus on technical standards for structured reporting is still missing.

4. The application of large language models may help structuring radiological reports.

5. Policy makers should incentivize the usage of structured radiological reporting.

**Graphical Abstract:**

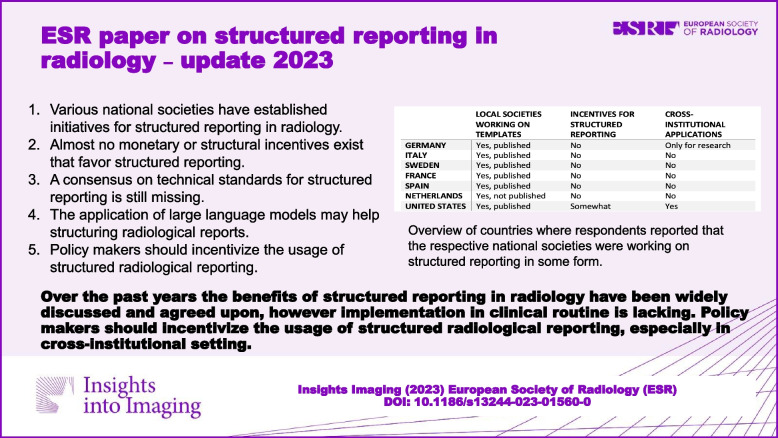

## Introduction

The topic of structured reporting is certainly not novel. Now almost a century ago, in the 1920s, Preston Hickey, a radiologist from Detroit, noticed that the variability in language and style prevented radiological reports to be used for further analysis and suggested that a more structured approach to radiological reporting may be the solution [1]. Fast forward 90 years and still structured reporting is not used in clinical routine—despite the fact that the overwhelming majority of clinicians indicate preferring structured, itemized reports [2]. Interestingly, in the same survey, radiologists were rather undecided if reports should be itemized and prose be rejected—with only half of the respondents agreeing with the respective statements [2]. Since then, another 10 years have passed, and while technology and digitization overall have certainly made impressive improvements, most workflows in radiological departments throughout Europe are still mere digital representations of paper-based workflows from decades ago—including the formatting of radiological reports as free, prose-like texts representing the radiologists “stream of consciousness” or verbal reasoning [3, 4].

Over the years, various studies have provided evidence that the implementation of structured reporting may be a key element to optimizing radiology’s contribution to patient outcomes and ensuring the value of radiologists’ work—as summarized in a recent systematic review as well as the European Society of Radiology’s (ESR) previous statement on this topic [5, 6]. Since then, various developments have taken place that will contribute to the unification of technical standards and the successful implementation of structured reporting in clinical routine.

This article aims to serve as an update to the ESR’s previous publication on the implementation of structured reporting, providing an overview on the current status of structured reporting as well as offering critical suggestions on future directions and policy development.

As there is some ambiguity in the usage of the term “structured reporting” in the literature, it is important to clarify that structured reporting in its truest sense can only be achieved when using dedicated IT solutions that allow for individual report items to be stored in a structured way that facilitates data mining (structured reporting level 2). In contrast, a radiological report can be highly standardized with regard to content and language (standardized reporting) or structured specific sections but without usage of a supporting IT tool (structured reporting level 1) [7].

## Overview of current technical standards

In the years since the publication of the last ESR white paper, most efforts towards structured reporting relied on the Integrating the Healthcare Enterprise Management of Radiology Reporting Templates profile (IHE MRRT). The Radiological Society of North America (RSNA) and the ESR joined forces and collaboratively worked on a collection of reporting templates with a joint Template Library Advisory Panel (TLAP). This led to the IHE MRRT profile becoming finally integrated in IHE’s radiological technical framework in early 2022 [8]. However, this profile was not widely adopted, and while the collection of templates is still accessible at https://radreport.org, it is very likely that no new templates will be published there. Instead, it seems more possible that other more advanced interoperable formats will be made available that e.g., allow for the incorporation of figures and links to imaging studies and basic image review capabilities—such as the IHE Interactive Multimedia Report [9]. These new approaches are in line with the preference of the so-called Fast Healthcare Interoperability Resources (FHIR), the adoption of which is fast growing and has been further fueled by various legislative measures making it a requirement in the United States’ healthcare system and the EU Health Data Space [10, 11]. The RSNA as well as the American College of Radiology have since shifted the focus to a more modular approach and will work towards incorporating common data elements (CDE—available at https://www.radelement.org) into FHIR (Fast Healthcare Interoperability Resources) or OMOP (Observational Medical Outcomes Partnership) compliant structures.

It remains to be seen which technical representation and implementation will be pursued in the end. Nonetheless, compared to previous years, substantial advancements have been made and the availability of interoperable formats will certainly help to convince vendors that clinically usable software solutions for structured reporting are needed.

## Overview of different national initiatives

It is clear that for a more widespread move towards structured reporting, it will be necessary to engage all stakeholders, such as information technologists and radiologists across borders. Unfortunately, information on different national implementation strategies for structured reporting is lacking. To get a better overview on the current status of structured reporting in different countries, the members of the ESR’s eHealth and Informatics Subcommittee performed a literature review and informally reached out to several representative radiologists as opinion leaders in their network around the world to collect responses to three key questions:

- *Are there any reporting templates that have been/are made available on a national level by the national radiology society or subspecialty societies?*
- *Are there any monetary or structural incentives to use structured reporting templates?*
- *Are there cross-institutional applications using structured reporting templates, e.g., for registries or research?*

Given the rather informal nature of this approach, there may well be initiatives and/or approaches to structured reporting that the respondents were not aware of. Similarly, all collected responses represent individual views, may only apply to the respective individual’s institution can therefore not be considered exhaustive. Nevertheless, the respondents’ qualitative answers can still provide valuable insights into the usage of structured reporting in different countries while allowing for patterns to be identified to help guide future developments.

### Europe

#### France

There are no general national initiatives for structured reporting in radiology, apart from the Women’s Imaging subspecialty society (SIFEM) who recently published report templates for both breast and gynecological imaging, including interventional [12]. However, there are no national incentives (particularly economic) and only individual initiatives of template-based reporting in institutions or in research contexts. An unofficial survey among radiologists of the executive committee of the oncologic imaging subspecialty society showed that all had a template or table used in their institution for RECIST evaluation, but none were cross-institutional. Only in the context of the COVID pandemic was a nationwide structured report proposed; however, the extent of its use was not evaluated at a national level.

#### Germany

Over the past years, the IT Subcommittee of the German Radiological Society (DRG) has been actively developing reporting templates on a national level and made them available on a dedicated website: www.befundung.drg.de. Currently, 26 report templates are available on the DRG’s website, most of which have been developed in consensus with other DRG subcommittees as well as other relevant scientific societies—e.g., in the case of the report templates for staging of pancreatic cancer, with the respective surgical and oncological societies, or in the case of the report templates for cardiac imaging with the respective cardiological societies [13–15]. While there are no monetary incentives to use reporting templates in Germany, the DRG’s reporting templates have recently been included in the national guidelines for diagnosis and treatment of pancreatic cancer, thus highlighting their value on a national level [16]. In contrast to this, respondents stated that despite templates being available and some institutions having bought structured reporting software, reporting in clinical routine is mainly done using free-text dictation—with some institutions being notable exceptions [17]. In research settings, first use-cases of cross-institutional applications using structured reporting are emerging. In the wake of the COVID-pandemic, a state-funded research project connecting all German university hospitals was established in which selected imaging data is collected alongside the corresponding structured reports for which dedicated templates have been developed [18].

#### Italy

A similar national initiative was established by the Italian Society of Medical and Interventional Radiology, where panels of expert radiologists developed various report templates for dedicated pathologies (including breast cancer, pancreatic cancer, lymphoma, neuroendocrine neoplasms and others) using the Delphi method and included them in the corresponding publications [19–26]. Despite these efforts, the contacted radiologists reported that there were no special incentives for using structured reporting templates and were not aware of cross-institutional applications.

#### Netherlands

Recently, the mammography section of the Dutch Society for Radiology (NVvR) has started working on a structured reporting template for mammography but has not yet published it. In parallel, other sections of the NVvR are working on standard reports, which do not directly refer to template-based reporting. Similar to other countries, no incentives are in place, and respondents were not aware of any cross-institutional applications of structured reporting.

#### Spain

The Spanish Radiological Society (SERAM) endorses the use of structured report templates, based on imaging modalities and anatomical parts together with disease-specific structured reports. The society highlights this endorsement by the many different papers on the 2022 annual meeting monograph published in the society’s journal [27]. Also, the Spanish Society for Neuroradiology (SENR) started an initiative to develop structured reporting templates. Currently, a dedicated report template for dementia assessment can be accessed on the SENR’s website [28]. Apart from that, the respondents were not aware of any initiatives on a national level and reported no incentives for the usage of structured report templates.

#### Sweden

Efforts to developing structured report templates have been coordinated between the Swedish Society of Radiology and the Swedish Colorectal Cancer Registry and as a result a dedicated structured report template was introduced [29]. As in other countries, no specific incentive is in place, and therefore—even for rectal cancer where a template is available—the majority of radiological reports are composed using free-text dictation.

#### Switzerland

While respondents were not aware of specific incentives for structured reporting in Switzerland, some institutions already implemented structured reporting in some form into their reporting workflows. The Swiss Society of Radiology (SGR/SSR) set up a working group on structured reporting to further coordinate the efforts on a national level and provide templates for download in all Swiss working languages [30]. Currently, reporting templates for a variety of examinations and clinical indications are available for download on their website—mostly in the form of word files with specific subheadings to better organize findings.

### Outside Europe

#### United States of America

While the RSNA’s reporting initiative [31] has been one of the first coordinated efforts to facilitate the adoption of structured reporting, the RSNA’s templates (www.radreport.org) are not widely used. Respondents stated that this is mainly due to vendors showing little interest in adopting interoperable standards such as IHE MRRT [8]. In contrast, on a departmental level, various structured reporting templates are used, mainly for cross-sectional imaging and interventional procedures [32]. In cases of departments spanning over multiple sites, e.g., multiple hospitals and outpatient centers, report templates are distributed across the enterprise. While on a national level the use of structured reporting templates is not monetarily incentivized per se, its usage has some advantages for institutions. In some cases where billing is tied to report completeness (e.g., to bill for a “complete abdomen ultrasound” explicit mentions of the inferior vena cava and the aorta must be included) or participation in quality improvement programs (e.g., requiring reporting of specific metrics or report items in breast and lung cancer) is mandatory, structured reporting helps ensure the necessary information are included in the radiologists’ final reports. Similarly, structured reporting can be used to facilitate data collection for registries, e.g., the American College of Radiology’s National Radiology Data Registry [33]. Interestingly, pathology departments are using structured reporting templates, too, and through its usage can receive credit towards their accreditation status.

#### United Kingdom

Given the RSNA’s templates’ availability, the need for a national initiative to develop structured reporting templates in the United Kingdom may always have been somewhat limited. Consequently, the respondents stated that there is no separate national initiative for structured reporting in the UK. Much as in other countries, it was stated that the usage of reporting templates is very variable and mostly a personal choice—only in very few instances are there individual departmental policies promoting their usage. The National Health Service does not require structured reporting to be used and does not provide any dedicated incentives. The respondents were not aware of any cross-institutional applications where structured reporting templates were used to facilitate data collection and exchange.

#### Turkey, Israel, and India

None of the contacted radiologists were aware of national initiatives regarding structured reporting in their respective countries, and neither are incentives offered nor are there any mandates in place. An initiative to promote structured reporting started in 2018 by the Turkish Ministry of Health and the Turkish Society of Radiology was not able to reach consensus for proposing reporting templates and ultimately only proposed report formats. Consequently, no reporting templates are available on a national level and reporting is done using free-text dictation. On a departmental level, some individual radiologists use reporting templates, e.g., for cardiac imaging, but this does not extend to cross-institutional applications.

#### Asia–Pacific

In a recently published position statement, the Asian Oceanian Society of Radiology (AOSR) recognizes the value of reporting templates and advocates that templates be individualized to accommodate heterogeneous access to resources within the diverse group of Asia Oceanian countries [34]. To further promote the usage of structured reporting templates, the AOSR aims to develop a platform (ASTeR—AOSR Structured Template Reporting) to host downloadable report templates that are reviewed and endorsed by the relevant subspecialty societies. The Asian Society for Abdominal Radiology is currently reviewing templates for rectal, liver, and prostate imaging which shall be published in a variety of working languages to reflect the diverse nationalities that make up the AOSR member societies.

## Challenges and future directions

### Workflow impact

As outlined in the ESR’s previous statement on structured reporting, one of the major hurdles to adoption of template-based reporting in clinical routine is the need to integrate it in such a way that the radiologist’s clinical workflow is not compromised. Depending on the local practice, this may mainly relate to the possibility of having voice-driven workflow within a structured reporting solution—or any other possibility of interacting with the report templates that does not require the radiologist to stop interacting with the images. Unfortunately, most of the currently available structured reporting software force the radiologist to look at the graphical interface instead of looking at the images to be interpreted [35]. Instead of concentrating on his main task, the radiologists’ attention is absorbed by handling the mouse and clicking on checkboxes and other graphical elements. While for the less experienced radiologists this may be a helpful option guiding them through the diagnostic workup, more experienced radiologists do not feel comfortable with such interfaces as they are perceived as a major obstacle to their productivity—despite many efforts from vendors to optimize such systems. Adding to this, the implementation of structured reporting might be perceived as oversimplifying in some cases or limiting the flexibility to express more complex findings. Some advocate that reporting templates should therefore contain several free text fields to accommodate for such situations, while others advocate that a modular approach with structured data entry possibilities added as needed would preferable. The most favorable approach may also vary depending on the clinical scenario; it will therefore be important for all stakeholders (radiologists, referring clinicians and others) to collaboratively develop reporting templates to maintain find a balance between what is clinically needed and helpful and does not impact or limit the radiologists’ workflow.

The effort of implementing structured reporting in clinical routine, however, can be expected to pay off. Most importantly, the possibility to leverage structured report data for secondary usage could help streamline clinical processes and support research. While currently many quality assurance initiatives rely on dedicated personnel extracting data from narrative radiology reports (e.g., for certification processes and quality assurance in specialized tumor centers or data entry into registries—e.g., such as the European Society of Cardiovascular Radiology’s CT/MR registry [36]), such processes could be easily be automated by accessing structured report data [37]. Similarly, follow-up recommendations—if captured in a structured format—could easily trigger automated workflows for scheduling or tracking as a solution to improve on the notoriously low follow-up rate of e.g., incidental pulmonary nodules [38]. Lastly, quantitative data obtained from the imaging study could automatically be integrated in a structured report, eliminating a potential source of error [39, 40].

Furthermore, in order to improve patient satisfaction and make radiological reports more accessible, structured reports could facilitate patients’ understanding of radiological reports—either through information like figures and video linked to the respective fields of a structured report template or the automated translation of report content to lay-language and other languages [41, 42]. Since patients usually have the right to full access of their medical reports and images, it would, however, be important that all versions of the radiological report—e.g., a patient-oriented, more understandable version and the original report mainly intended for communication with the referring physician—remain ultimately equivalent in content.

Lastly, it should be noted that the impact of structured reporting on radiologist’s visual search patterns has not yet been scientifically evaluated. While it is plausible that the structure provided by reporting templates could help guide a systematic interpretation of the imaging data ensuring that no potentially relevant part is missed (especially for less experienced trainees), it could also be argued that such guidance narrows the radiologists’ attention down to what is included in the template and over time could lead to a deterioration in the radiologists’ critical thinking and adaptability. Institutions introducing structured reporting on a larger scale should be aware that these effects could occur and if possible gather evidence for subsequent analysis.

### Structured reporting and artificial intelligence

Many potential synergies come to mind when considering structured reporting in the context of the recent developments in artificial intelligence. The most trivial of those being that structured report data would substantially facilitate the development of AI models as report data could more easily be used as label data for training and validation [43, 44]. But more importantly with AI tools being increasingly used in clinical practice, structured reporting could offer a possibility to integrate AI results more seamlessly into the radiological report. The communication of AI results to a reporting system can be done in several ways—e.g., using DICOM SR objects. However, more recently, IHE has published two dedicated interoperability profiles: AI Results (AIR) and AI Workflow of Imaging (AIW-I) [45, 46]. While AIR more specifically focuses on interoperability between AI tools and reporting solutions describing how to efficiently integrate AI results into the reading environment, AIW-I aims to describe how a meaningful and efficient workflow in the interaction between AI tools, PACS, and reporting solutions can be ensured.

On the other hand, the recent advancements in natural language processing—most notably in the form of ChatGPT, GPT-4, and similar large language models—might present a solution to the challenges in the workflow integration aspects of structured reporting [47]. In the past years, some efforts have already been made to extract information from unstructured reports and make this data available for further analysis [48–52]. Combining modern transformer-based models with domain-specific, radiological text data showed even more promising results [53, 54]. If these technologies could be successfully combined with structured reporting templates and speech recognition, it would seem feasible to maintain the usual workflow with free-text dictation while those language models would extract the relevant data and fill the template accordingly—possibly even prompting the reporting radiologist to provide information on items included in the report template but not yet mentioned in the dictation [55, 56]. The use of such chatbots and natural language processing may revolutionize the way radiological reporting and other medical writing tasks will be done in the future.

Similarly, this technology could also enhance the value of previously unstructured reports by enabling their utilization and analysis. This would not only maximize the value of retrospective data but also grant added flexibility to radiologists by allowing them to work with less rigid report structures for present and future cases, too. Real-time structuring of information contained in the radiology reports would empower clinicians by providing readily accessible and structured information, facilitating statistical analysis, and enabling real-time integration with AI systems. These developments may hold the potential to revolutionize the radiological workflows by unlocking the potential of both historical and current reports for improved patient care and efficient data-driven decision-making.

### Summary and future plans

Even though there is growing evidence that a more structured approach to radiology reporting would beneficial, adoption in clinical routine is still lacking. As stated in the previous position paper, it is the ESR’s conviction that structured reporting will be important in providing the best service to referring physicians and patients [5]. However, to best convince stakeholders that in fact structured reporting can have a positive impact on patients, more outcome-oriented research is needed. Lastly, the possibility to reuse report data from clinical routine will facilitate not only research but could also offer new possibilities to optimize clinical workflows. The radiology report will therefore be one of the building blocks in the transition to value-based radiology [57, 58].

Even though it is still unclear which will be the way forward after the RSNA suspended its efforts towards www.radreport.org, it is clear that radiological societies like the ESR and the RSNA will lead the way in pushing for more widespread use of structured reporting. While the technical integrations into the radiologists’ workflow will need to be provided by industry vendors, some form of incentive probably needs to be established to really get the fusion reactor going [3]. The ESR will continue its efforts facilitate collaboration between its subspecialty societies as well as its members’ national societies—ideally in some cases leading to a European consensus on reporting templates, report elements, and artificial intelligence best practices.

## Data Availability

Not applicable.

## Abbreviations

AIR: AI Results
AIW-I: AI Workflow of Imaging
AOSR: Asian Oceanian Society of Radiology
CDE: Common data elements
DRG: Deutsche Röntgengesellschaft (German Radiological Society)
FHIR: Fast Healthcare Interoperability Resources
GPT: Generative pre-trained transformer
IHE: Integrating the Healthcare Enterprise
MRRT: Management of Radiology Reporting Templates
NVvR: Nederlandse Vereniging voor Radiologie (Dutch Society for Radiology)
OMOP: Observational Medical Outcomes Partnership
RSNA: Radiological Society of North America
SENR: Sociedad Española de Neuroradiología (Spanish Neuroradiological Society)
SERAM: Sociedad Española de Radiología Médica (Spanish Radiological Society)
SGR/SSR: Schweizerische Gestellschaft für Radiologie/Société Suisse de Radiologie/Swiss Society of Radiology
SIFEM: Société d'Imagerie de la Femme (French Women’s Imaging subspecialty society)
TLAP: Template Library Advisory Panel
